# ICD-11 extension codes support detailed clinical abstraction and comprehensive classification

**DOI:** 10.1186/s12911-021-01635-2

**Published:** 2021-11-09

**Authors:** Saskia E. Drösler, Stefanie Weber, Christopher G. Chute

**Affiliations:** 1 Faculty of Health Care, Niederrhein University of Applied Sciences, Reinarzstr 49, 47805 Krefeld, Germany; 2 Federal Institute for Drugs and Medical Devices, Kurt-Georg-Kiesinger-Allee 3, 53175 Bonn, Germany; 3 Schools of Medicine, Public Health, and Nursing, Johns Hopkins University, 2024 E Monument St, Suite 1-200, Baltimore, MD 21287 USA

**Keywords:** Classification, International Classification of Diseases, ICD11, Extension codes

## Abstract

**Background:**

The new International Classification of Diseases—11th revision (ICD-11) succeeds ICD-10. In the three decades since ICD-10 was released, demands for detailed information on the clinical history of a morbid patient have increased.

**Methods:**

ICD-11 has now implemented an addendum chapter X called “Extension Codes”. This chapter contains numerous codes containing information on concepts including disease stage, severity, histopathology, medicaments, and anatomical details. When linked to a stem code representing a clinical state, the extension codes add significant detail and allow for multidimensional coding.

**Results:**

This paper discusses the purposes and uses of extension codes and presents three examples of how extension codes can be used in coding clinical detail.

**Conclusion:**

ICD-11 with its extension codes implemented has the potential to improve precision and evidence based health care worldwide.

## Background

The International Classification of Diseases—11th revision (ICD-11) has a linearization version for Mortality and Morbidity Statistics. This is a tool that can describe the clinical status of a patient in a structured manner [1]. It succeeds ICD-10, which was released more than three decades ago. In the meantime, demands for detailed information on the clinical history of a morbid patient, regardless of point of care (e.g. inpatient or outpatient) have increased. Rising demands on clinical documentation beyond disease classification, such as histopathology, epidemiology, quality and safety, clinical administration, severity, anatomic detail, and research purposes underpin the need for a more detailed disease classification system. Furthermore, the rapid development of IT infrastructure including logical and sanctioning heuristics along with data storage and computational capacity supports the use of a much broader set of rules and coded clinical concepts that together permit rich clinical descriptions. An overview and details on the architecture of ICD-11 are provided in other articles in this series.

## Main text

ICD-11 has implemented an addendum chapter X called “Extension Codes”. This chapter for optional use contains numerous codes, each starting with an “X”, determined for use together with a “stem code”. Extension codes are supposed to add relevant information to stem codes. Stem codes can stand alone whereas extension codes cannot. Extension codes always follow stem codes and may not occupy the first position in a code cluster. Using clusters of more than one ICD code for classification of a clinical concept is called postcoordination—a feature that allows a much more detailed capture of clinical information. Postcoordination is described in detail in a dedicated article elsewhere in this series.

Extension codes contain information on concepts including disease stage, severity, histopathology, medicaments, and anatomical details. When linked to a stem code representing a clinical state, the extension codes add significant detail and allow for multidimensional coding. This approach produces longer code strings, but in doing so, substantially reduces the total number of stem codes needed in other ICD-11 chapters.

According to the ICD-11 Reference Guide [2], the hierarchically rendered extension codes listed in addendum chapter X are not a classification. Entities are not mutually exclusive. However, various non-hierarchical clinical classifications such as tumour spread, the New York Heart Association Functional Classification (NYHA, e.g. *XS6B NYHA Class II—Slight limitation of physical activity*) [3], or a 14 item clinical staging scale are implemented in chapter X. Levels of hierarchy may vary from just one in the section “Capacity or context “ up to eight in the section “Anatomy and topography”.

There are two types of extension codes. A type 1 code adds detail on an entity or disease coded from ICD-11 chapter 1–26. Type 2 extension codes qualify a diagnosis and can be applied to codes from any chapter. Diagnosis qualification means an additional detailed description of a coded disease (for diagnosis type and diagnosis timing, Table 2) and is a new feature of ICD-11. It significantly enriches clinical content and supports administration [4] as well as quality and safety management [5]. Table 1 depicts all headline categories from the extension codes chapter and their dedicated type. Furthermore, related to each of the head categories, Table 1 shows a selected example in hierarchical order. Table 2 shows the full list of codes available for diagnosis description.

**Table 1:** Head categories listed in the ICD-11 extension codes chapter and selected examples in hierarchical order (09/30/2021)

Head categories: all parents in ICD-11 chapter X	Type of Extension codes	Examples in hierachical order			
		Subheading (Parent)	Subheading (Parent)	Subheading (Parent)	Most detailed category
Severity Scale Value	1	Disease Specific Severity Scale Value	Tumour spread staging scale value		XS4P Stage II
Temporality	1	Duration of pregnancy			XT4J Duration of pregnancy 26–33 completed weeks
Aetiology	1	Infectious Agents	Virus	XN83D Coronavirus XN109 SARS-CoV-2	XN8V6 SARS-CoV-2 Delta
Topology Scale Value	1	Laterality			XK9K Right
Anatomy and topography	1	Functional anatomy	Digestive system	XA0KT3 Biliary tract	XA8KL9 Gallbladder
Histopathology	1	Adenomas and adenocarcinomas	Adenomas and adenocarcinomas, malignant		XH74S1 Adenocarcinoma, NOS
Dimensions of injury	1	Whether fracture is open or closed			XJ44E Closed fracture
Dimensions of external causes	1	Aspects of transport injury events	Mode of transport of person injured in transport event	XE7NK Motorcycle as mode of transport of person injured in transport related event	XE2J1 Moped as mode of transport of person injured in transport related event
Consciousness	1	Pupil reaction score			XC5Y Neither pupil reacts
Substances	1	Medicaments	Analgesics, antipyretics and anti-inflammatory drugs	Acetylsalicylic acid and other salicylates	XM4G06 Acetylicsalicylic acid
Diagnosis code descriptors^#^	2	Diagnosis certainty			XY7Z Provisional diagnosis
Capacity or context*	2				XX2QG9 Condition of the fetus and newborn reported in the context of the mother
Health devices, equipment and supplies	1	Respiratory and anaesthesia devices	XD5GF6 Respiratory masks and balloons, single-use and reusable	XD3W67 Air/oxygen masks and nasal cannulas	XD0VQ3 Air/oxygen masks

^#^For details see Table 2

^*^This parent has only one detailed category

**Table 2:** All detailed codes for diagnosis description (09/30/2021)

Subheading (Parent)	
Discharge diagnosis types	XY0Y Main condition
	XY7B Main resource condition
	XY6E Initial reason for encounter or admission
Diagnosis timing	XY6M Present on admission
	XY69 Developed after admission
	XY85 Uncertain timing of onset relative to admission
Diagnosis timing in relation to surgical procedure	XY9U Preoperative
	XY9N Intraoperative
	XY7V Postoperative
Diagnosis method of confirmation	XY3B Diagnosis confirmed by laboratory examination
	XY0E Diagnosis confirmed by serology
	XY9Q Diagnosis confirmed by histology
	XY8K Diagnosis confirmed by genetics
	XY9R Diagnosis confirmed by imaging
	XY19 Diagnosis confirmed by microscopy
	XY0K Diagnosis confirmed by culture
Diagnosis certainty	XY7Z Provisional diagnosis
	XY75 Differential diagnosis
Obstetrical diagnosis timing	XY3K Delivered with or without mention of antepartum condition
	XY8Q Delivered, with mention of postpartum condition
	XY8U Antepartum condition or complication
	XY9P Postpartum condition or complication
	XY9S Unspecified as to episode of care, or not applicable
Encounter descriptors	XY18 Initial encounter
	XY8S Subsequent encounter

While compiling extension codes, strong consideration was given to aligning the content of the extension code chapter with existing systems in place like the TNM-classification [6] and the histopathology from ICD-Oncology [7] for tumor description or even the substances in the Anatomical Therapeutic Chemical Classification System ATC [8]. Where a coder previously had to use two or more coding systems, now all information can be found within ICD-11, and in cross-referencing to other clinical description systems, good continuity of health information is achieved. Interoperability among systems is fostered, while historical use of multiple systems is recognised and enabled.

### Purposes and use of extension codes

There are several different perspectives for secondary use of coded health information. Epidemiological surveillance focuses on stem codes representing certain diseases or conditions. Monitoring of quality and safety, meanwhile, benefits from extension codes that provide information on diagnosis timing, or disease stages for risk adjustment. Case Mix based reimbursement systems or risk equalization schemes may profit from clinical details mediated by extension codes fostering differentiation of statistical modelling. The following examples provide a selection of clinical cases applying entities from the extension codes chapter X. Clusters of stem codes are separated by a forward slash (/). An ampersand (&) is used for clustering a stem code and one or more extension codes. If a case involves different clinical concepts, they are clustered separately by convention [2]. For coding of the examples below, the WHO ICD-11 Coding Tool [9] was applied.

All examples are constructed and neither correspond to real clinical cases nor to any datasets.

#### Example 1

A male patient, 75 years, is admitted to hospital for an elective endovascular repair of an infrarenal aortic aneurysm detected by ultrasound earlier. He has a hypertensive heart disease associated with mild chronic left ventricular failure. His ejection fraction is unknown but his physical activity is slightly reduced according to NYHA 2. Postoperatively he presents a deep wound infection at the access site (left groin), reason unclear. Microbiological proof of a Methicillin resistant Staphylococcus aureus.

- BD50.4Y Other specified abdominal aortic aneurysm &
- **XA2LN9** Infrarenal abdominal aorta &
- **XY9R** Diagnosis confirmed by imaging&
- **XY0Y** Main condition

- BA01 Hypertensive heart disease /
- BD11.Z Left ventricular failure, unspecified &
- **XS6B** NYHA Class II—Slight limitation of physical activity &
- **XY6M** Present on admission.

- NE81.21 Deep incisional site infection /
- MG51.00 Methicillin resistant Staphylococcus aureus /
- PK80.91 Vascular procedure associated with injury or harm, percutaneous approach /
- PL11.Z Unspecified mode of injury or harm associated with a surgical or other medical procedure &
- **XA0NH8** Iliac region &
- **XK8G** Left &
- **XY3B** Diagnosis confirmed by laboratory examination &
- **XY7V** Postoperative.

This example contains three clusters derived from three medical problems the patient presented:

Reason for admission: BD50.4Y & **XA2LN9** & **XY9R** & **XY0Y.**
Chronical diseases: BA01 / BD11.Z & **XS6B** & **XY6M.**
Postoperative complication: NE81.21 / MG51.00 / PK80.91 / PL11.Z & **XA0NH8** & **XK8G** & **XY3B** & **XY7V**

Extension codes contribute to an enhanced level of detail. From the clinical perspective, a distinct anatomical location of both, operation site and postoperative wound infection, and the specific severity scale value (NYHA) are registered. Diagnosis related information such as timing and confirmation mode and information about the causative organism support quality management. Regarding the three-part model for capturing safety events, harm and cause are applied. As the mode of harm remains unclear, an unspecified residual category is applied to complete the three-part model.

For comparison, the information on this case coded in a far more limited way in ICD-10 [10] is presented:

- I71.4 Abdominal aortic aneurysm, without mention of rupture.
- I11.0 Hypertensive heart disease with (congestive) heart failure.
- T81.4 Infection following a procedure, not elsewhere classified.
- B95.6 Staphylococcus aureus as the cause of diseases classified to other chapters.
- U82.1 Resistance to methicillin.

#### Example 2

A female patient, 45 y, is admitted to the emergency room with a traumatic brain injury after she fell from a horse (leisure activity in a forest). She is unconscious but opens her eyes with both pupils reacting and shows specific moves to local stimulation. No verbal output. CT scan shows a diffuse edema of the brain. There are no further injuries. Her relative reports that she has a history of breast cancer (right side); details on the type of cancer are unknown. Incidentally, an abdominal ultrasound detected a mass in her liver (left lobe). After her cerebral recovery an image guided biopsy of the mass in her left lobe of the liver was performed. The histopathological analysis revealed a metastasis of her invasive ductal carcinoma (histological grade 2).

- NA07.20 Diffuse traumatic oedema /
- NA07.09 Loss of consciousness, duration unspecified or unknown due to lack of information &
- **XY9R** Diagnosis confirmed by imaging &
- **XC5K** Both pupils react &
- **XC3H** One or both eyes open to painful or noxious stimulation &
- **XC6J** Localizes response to painful or noxious stimulation &
- **XC8U** No verbal output even with painful or noxious stimulation &
- **XY0Y** Main condition

- PA1E Unintentional land transport nontraffic event injuring a rider of an animal&
- **XE5C9** Other specified sports and exercise during leisure time &
- **XE6AV** Forest&
- **XE1LH** Type of sport or exercise activity, trail or general horseback riding.

- 2D80.0 Malignant neoplasm metastasis in liver &
- **XA5766** Left lobe of liver &
- **XH7KH3** Infiltrating duct carcinoma, NOS &
- **XS58** Grade II &
- **XY9Q** Diagnosis confirmed by histology /
- 2C6Z Malignant neoplasms of breast, unspecified &
- **XK9K** Right.

Code cluster on the injury and external cause:

- NA07.20 / NA07.09 & **XY9R** & **XC5K** & **XC3H** & **XC6J** & **XC8U** & **XY0Y** /
- PA1E & **XE5C9** & **XE6AV** & **XE1LH**

Code cluster on the malignant neoplasm metastases:

- 2D80.0 & **XA5766** & **XH7KH3** & **XS58** & **XY9Q** / 2C6Z & **XK9K**

**Fig. 1:**
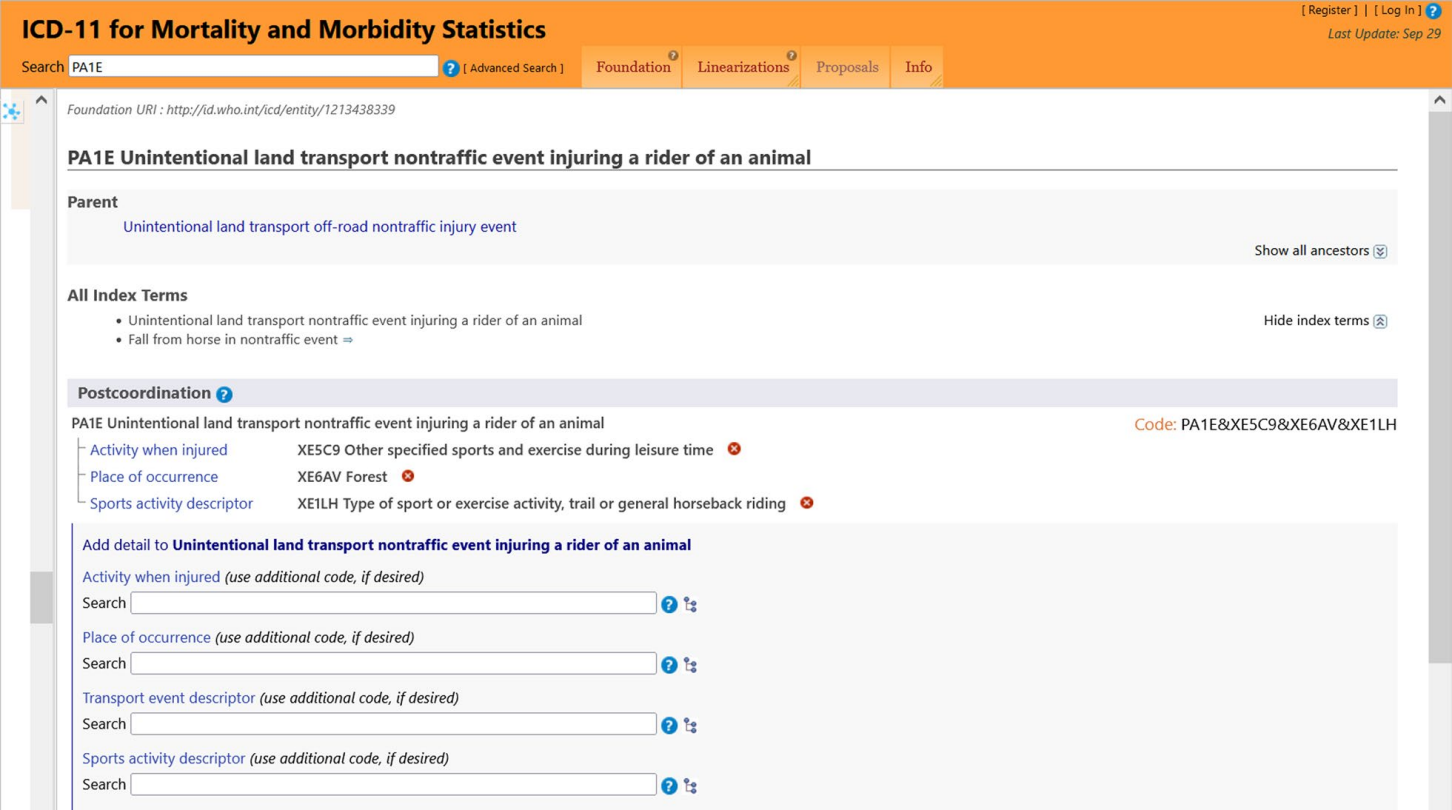
Extension codes for external cause specification and automatic postcoordination

**Fig. 2:**
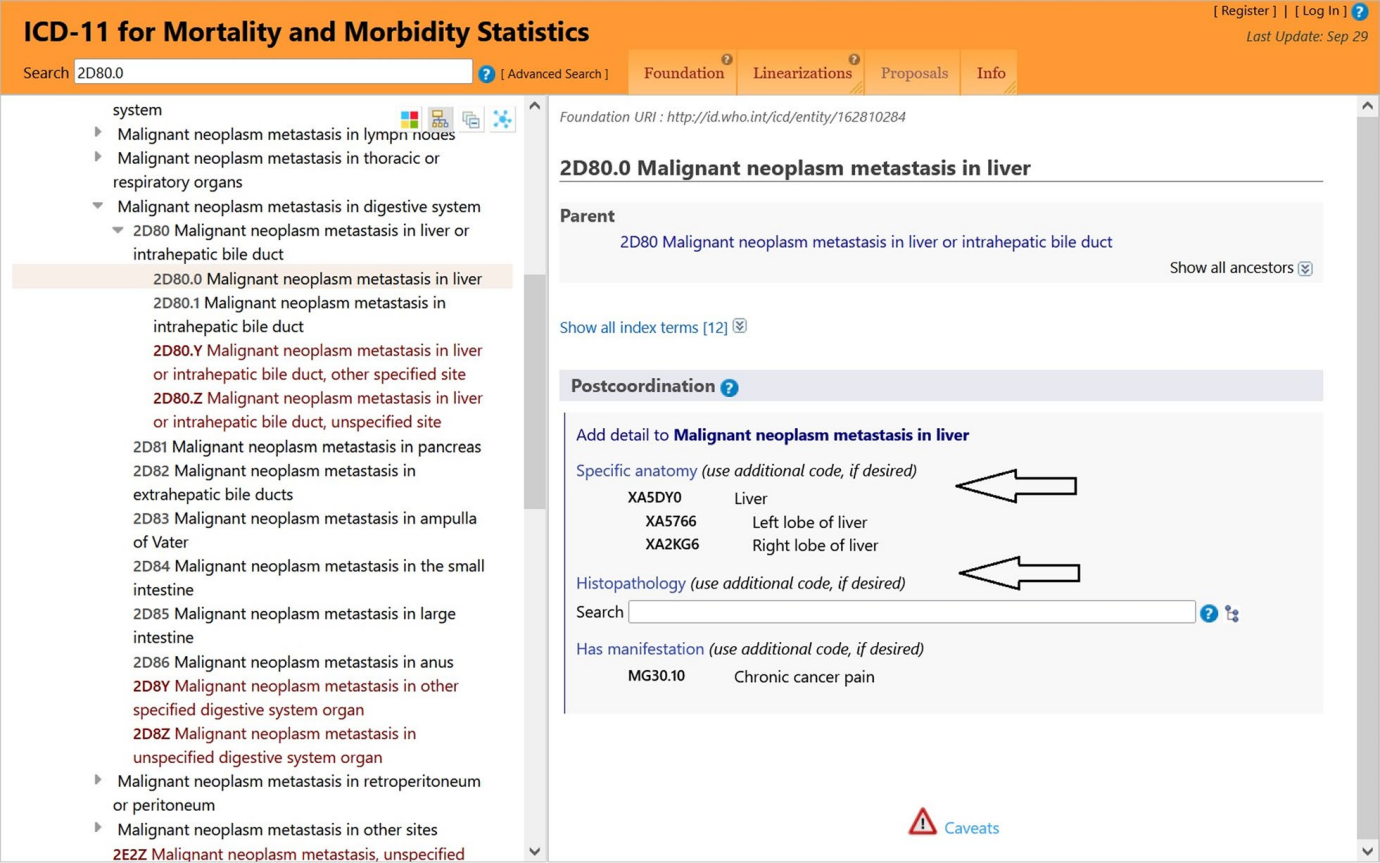
Extension codes offered by the system for specification of a clinical diagnosis

This example represents the clinical disciplines of trauma and oncology and contains two clusters. The fist cluster on the injury and external causes shows entities from the Glasgow Coma Scale axes applied in patients with brain injuries. Several codes allow detailed information on the course of injury, shown in Fig. 1. First, the ICD 11 coding tool supported the detection of the related stemcode using the entry terms “horse fall”. Second, chapter X provides numerous extension codes in the sections “Dimensions of external causes” to capture mechanisms of accidents precisely. The postcoordination tool implemented in the ICD-11 software is a substantial benefit. In the example, the software led us to the following parent dimensions: “Activity when injured (XE5C9)”, “Place of injury occurrence (XE6AV)”, and “Sports activity descriptor (XE1LH)”. A “Transport event descriptor”, as presented in Fig. 1, was omitted, because no counterpart was involved in the accident. Not all of these codes add substantial clinical information. For statistical reasons (statistics on causes of accidents) or causality insurance, they might be relevant. Applying Chapter X in the second cluster, oncological entities related to a tumor diagnosis from Chapter 02 are captured in detail. Figure 2 shows, that the ICD-11 software automatically reminds the user of postcoordination and offers a code selection from Chapter X. Implementation of histopathologic tumor cell morphology according to ICD-O as well as histological grading and clinical staging adds significant information on the neoplasm.

#### Example 3

A female patient, 41 y, is readmitted to the hospital (emergency room) presenting severe abdominal pain. She has a marked obesity (BMI 38 kg/m^2^, according to Obesity class II WHO). A week before she had an open cholecystectomy due to cholecystitis from stones in gallbladder. Because of intraoperative bleeding, a conversion from laparoscopic to an open access to the gall bladder was necessary. The thorough examination including imaging revealed an intraabdominal foreign body. A surgical sponge causing a localized peritonitis was removed by laparotomy. No further problems during recovery. The protocols on team time out or counting results from the first operation are untraceable.

- DC50.14 Secondary peritonitis due to other diseases or agents /
- PL11.3 Foreign body accidentally left in body, as mode of injury or harm /
- PK80.30 Gastrointestinal, abdominal, or abdominal wall procedure associated with injury or harm, open approach &
- **XY9R** Diagnosis confirmed by imaging &
- **XY7V** Postoperative &
- **XY0Y** Main condition &
- **XY8S** Subsequent encounter.

- 5B81.01 Obesity in adults &
- **XS6N** Obesity class II BMI 35.0–39.9

This example is related to quality and safety. The three-part concept of harm, mode, and cause (expanded on in another article in this series) is applied [11], however, in this case a cause, possible failure of team time out postoperatively, cannot be assigned. An addition of an extension code provides information on the anatomic location of the foreign body. Code XY8S “Subsequent encounter “ is related to the parent “diagnosis code descriptors” (see Table 2) and provides useful information on the fact that the patient had been treated earlier in this institution. Numerous severity scales are offered in chapter X: Obesity classes according to the Adult Nutritional Status Scale Value are listed here and used in this example. A precise description of the obesity, which is a risk factor for retained instruments and sponges, adds relevant clinical information [12].

## Discussion

The clinical examples above demonstrate how ICD-11 codes from chapter X support highly detailed documentation coding. Even though the use of multiple codes for each situation seem to be more complicated to assign, it is aided or fully generated through electronic support such as the ICD-11 browser and the Coding tool.

Substantial field testing of ICD-11 has been undertaken by the WHO and its various network collaborating centers, throughout the development stages of ICD-11. These have demonstrated considerable acceptance and understanding among health information stakeholders who are involved in generating current coded health data using ICD-10. The field testing done has revealed that the ICD-11 coding tool is powerful in assisting coders in generating code clusters, guiding post-coordinated selection of relevant extension codes that relate to a stem code clinical concept [13, 14].

### Granularity of coding

Faced with such rich clinical coding content, the coder must balance which piece of information is important to represent the clinical condition as well as fulfil purposes of administration and quality and safety management. The level of granularity will depend on the purpose of use. Clinical information for case mix assignment need less granularity in histopathological details than cancer registries. With collection of information in a clustered form, electronic use of the elements of the cluster according to the use case will be possible.

Extension codes always provide additional information to a disease, disorder, or injury coded from another ICD-11 chapter. Their assignment depends on the individual motivation of the person coding, and the regulatory/administrative/clinical needs of the health system in which coding will occur. International recommendations or national documentation rules will determine the guidelines for correct use of these codes for local, national, and international purposes. Example 2 is presented, intentionally in this article, as a very complex case where heavy use of extension codes is possible and potentially valuable. Indeed, various extension codes such as the different severity scales and dimensions of injury significantly refine clinical information and could have economic relevance. Furthermore, distributed clinical information systems have potential to support coding directly at the point of care.

Overwhelming granularity of coding can be regarded as a limitation. Detailed knowledge about availability of specific codes is necessary when the coding tool is not used. Computer-assisted code assignment is imperative for high quality coding as code abstraction from printed materials is time consuming and was not considered conceptualizing ICD-11.

Despite extensive electronic support of the coding process, associated training will be fundamental to secure consistency in medical chart abstractions.

Reproducibility in the setting of human coding, where selection among many possible modifiers may be subjective or at least influenced by experienced patterns and case mix, can cause limitations.

### Secondary use of coded data requires distributed data systems

In evaluation and secondary use of the data, it will now be possible to extract information from code clusters. Furthermore, the opportunities for data-driven discovery and best evidence generation in the spirit of the Learning Health System [15] may be among the strongest contributions of this next-generation ICD coding system. The granular histopathological description of a neoplasm, for example, is certainly useful from the perspective of cancer epidemiology and cancer registries but this use requires a strong infrastructure of linked data systems to share the coded data accordingly. Codes could be assigned in pathology labs and automatically derived from pathology information systems at the time of clinical coding. The destination route should be a multiple use of information coded just once.

In this regard, ICD-11 is designed for predominant electronic use, where clustering of codes and use of extension codes are strongly supported. IT-systems need to automatically offer relevant extension codes related to the selected starting code of a cluster. This feature, as implemented in the ICD-11-browser, will have an overwhelming benefit and supports detailed abstraction of clinical cases in different respects demonstrated above without increasing the burden on the clinical coder at the point of care.

Inclusion of aspects historically used in parallel, like the TNM-classification or information on the diagnosis certainty, will allow the coder to assign all information at once without using different systems. Still, extraction of relevant information according to the parallel systems like histopathology information for cancer registries will be possible without hindering backwards compatibility.

As ICD-11 is not in use yet, it is too early to present hard proof on superiority of ICD-11 relative to earlier versions of the International Classification of Diseases. However, as ICD-11 is implemented by countries over coming years, empirical studies based on extensive datasets will become possible to formally assess the richness of clinical information in coded health data systems.

## Conclusion

ICD-11 with its extension codes implemented has the potential to improve precision and evidence based health care worldwide when abstracted information will be shared among clinicians, caretakers, managers, and researchers.

## Data Availability

Not applicable.

## Abbreviations

ATC: Anatomical Therapeutic Chemical Classification System
ICD: International Classification of Diseases
ICD-10: International Classification of Diseases, 10th revision
ICD-11: International Classification of Diseases, 11th revision
ICD-O: International Classification of Diseases for Oncology
IT: Information Technology
NYHA: New York Health Association Functional Classification
TNM: T, extent of the primary tumour; N, absence or presence of the disease into the lymph nodes; and M, absence or presence of distant metastasis
WHO: World Health Organization
